# Customized Polymethyl Methacrylate Implants for the Reconstruction of Craniofacial Osseous Defects

**DOI:** 10.1155/2014/358569

**Published:** 2014-06-30

**Authors:** André Luis Fernandes da Silva, Alexandre Meireles Borba, Niverso Rodrigues Simão, Fábio Luis Miranda Pedro, Alvaro Henrique Borges, Michael Miloro

**Affiliations:** ^1^Post-Graduation Department, Faculty of Dentistry, University of Cuiaba (UNIC), 3100 Manoel Jose de Arruda Street, 78065-480 Cuiaba, MT, Brazil; ^2^Department of Oral and Maxillofacial Surgery, College of Dentistry, University of Illinois (UIC), 801 South Paulina Street, Chicago, IL 60612-7211, USA; ^3^Division of Dentistry, University General Hospital, 2101 Treze de Junho Street, 78025-000 Cuiaba, MT, Brazil

## Abstract

Craniofacial defects represent alterations in the anatomy and morphology of the cranial vault and the facial bones that potentially affect an individual's psychological and social well-being. Although a variety of techniques and restorative procedures have been described for the reconstruction of the affected area, polymethyl methacrylate (PMMA), a biocompatible and nondegradable acrylic resin-based implant, is the most widely used alloplastic material for such craniomaxillofacial reconstruction. The aim of this study was to describe a technique for aesthetic and functional preoperative customized reconstruction of craniofacial bone defects from a small series of patients offered by the Brazilian public health system. Three adult male patients attended consultation with chief complaints directly related to their individual craniofacial bone defects. With the aid of multislice computed tomography scans and subsequent fabrication of the three-dimensional craniofacial prototype, custom-made PMMA implants were fabricated preoperatively. Under general anesthesia, with access to the craniofacial defects with a coronal approach, the PMMA implants were adapted and fixated to the facial skeleton with titanium plates and screws. Postoperative evaluation demonstrated uneventful recovery and an excellent aesthetic result. Customized prefabricated PMMA implants manufactured over the rapid prototyping models proved to be effective and feasible.

## 1. Introduction

Craniofacial defects represent alterations in the anatomy and morphology of the cranial vault and the facial bones as a result of trauma, tissue necrosis associated with infections, or sequel following surgical procedures [1]. Craniofacial traumas from sport-related accidents, falls, physical assault, or, in particular, automobile accidents are associated with a high incidence of craniofacial defects by maxillofacial bone fracture caused by the trauma itself or bone loss caused by a neurosurgical craniotomy performed for intracranial decompression [2]. Such defects are not always corrected immediately due to the patient's compromised systemic condition(s) that may contraindicate immediate repair; this creates aesthetic and functional impairments of the facial skeleton that will need to be corrected at a later period of time [3]. Regardless of the etiology, these defects potentially affect an individual's psychological and social well-being [4].

Reconstruction of craniofacial defects requires a combination of function and aesthetic repair principles [3]. A variety of techniques and restorative procedures have been described for this purpose, including autogenous or allogeneic bone grafting, as well as the use of alloplastic materials that can be molded during, or prior to, surgical reconstruction [3, 4]. Each of the possible options has advantages and disadvantages in its use [5]. Autogenous bone grafting remains the first choice for reconstruction of bone defects, but often the patient refuses a second surgical site with resultant morbidity to obtain bone or the amount of bone available is insufficient; for such cases, alloplastic implant materials are used [3, 6]. Currently, the most widely used alloplastic material for these craniomaxillofacial reconstructions is polymethyl methacrylate (PMMA), a biocompatible and nondegradable acrylic resin-based implant, which can be fabricated prior to surgery, with the advantages of reduced surgical time, the use of a simple technique, and excellent long-term aesthetic results [3, 5, 7].

The construction of the PMMA implant can be performed during surgery, or even preoperatively, with the aid of three-dimensional (3D) reconstruction [1, 2, 4]. However, such technology is not always available depending upon the specific location, or the high cost of design and materials might preclude its use. Considering the cost-free availability of 3D reconstruction of the craniofacial skeleton for patients of the Brazilian public health system by the* Centro de Tecnologia da Informação Renato Archer* (CTI), the aim of this study was to describe a technique for aesthetic and functional preoperative customized reconstruction of craniofacial bone defects from a small series of patients.

## 2. Cases Report

Three adult male patients attended consultation at the Department of Oral and Maxillofacial Surgery at the General Hospital of Cuiaba, which is affiliated with the University of Cuiaba, MT, Brazil, with chief complaints directly related to their individual craniofacial bone defects.

### 2.1. Case 1

**Figure 1:**
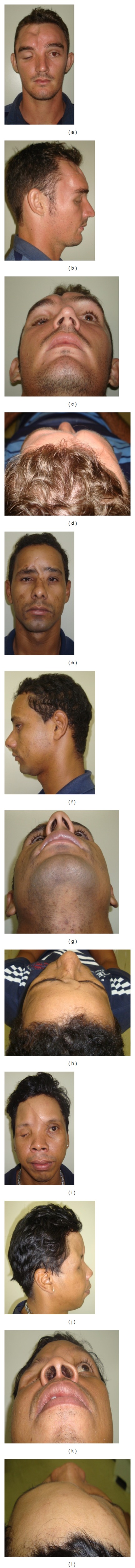
Clinical preoperative evaluation.

A 25-year-old male patient reported a work-related accident 18 months ago. At the time, he underwent decompressive craniectomy and remained hospitalized at an intensive care unit for a period of two months. Following hospital discharge, due to cosmetic and functional concerns about his craniofacial defect, the patient sought consultation and evaluation from our department. Physical examination showed a significant bony depression at the right frontoorbital region associated with right eye enophthalmos, orbital vertical dystopia, and right eyelid ptosis (Figures 1(a)–1(d)).

### 2.2. Case 2

A 30-year-old male patient was the victim of an altercation involving a large animal about five years ago. At the time of the accident, he was primarily treated at his hometown and referred to our department for evaluation and treatment of complex facial bone fractures. Several weeks later, the patient underwent reconstructive surgery for open reduction and internal fixation of the comminuted fractures of the frontal bone. At a late postoperative evaluation, it became evident that the patient presented with resorption of the anterior wall of the frontal sinus where titanium plates had been used for fixation of the fractures. During the next four years, the patient was noncompliant with regular follow-up visits and appeared in our clinic with a chief complaint of a significant frontal bone defect (Figures 1(e)–1(h)).

### 2.3. Case 3

A 33-year-old male patient was the victim of a motor vehicle accident when he was 18 years of age. At that time, he underwent decompressive craniectomy, exenteration of the right eyeball due to extensive injury, and tracheotomy while being hospitalized in the intensive care unit for a period of two months. The patient remained untreated due to the extensive distance from his hometown to the nearest health services facility until he was referred to our department for evaluation and management. Physical examination revealed a significant depression involving the right frontonasoorbital region (Figures 1(i)–1(l)).

### 2.4. Preoperative Planning

**Figure 2:**
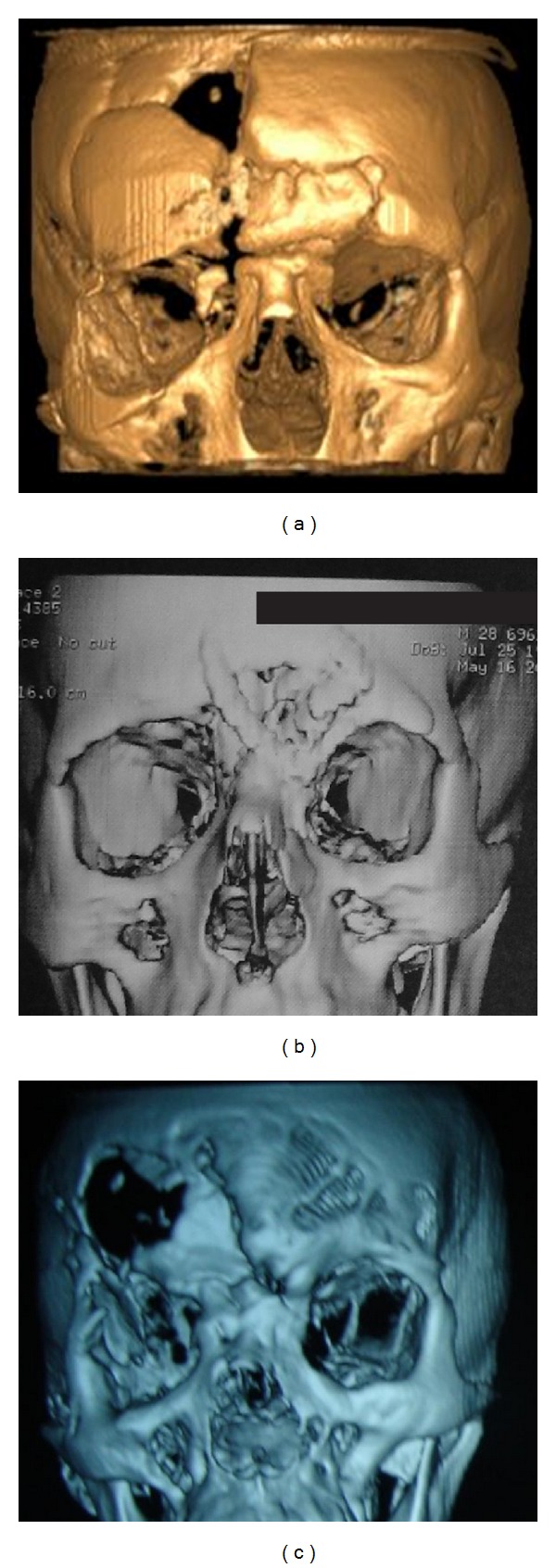
Preoperative 3D CT scans for Cases 1, 2, and 3 ((a), (b), and (c), resp.).

In order to obtain a precise diagnosis and perform surgical planning for reconstruction of the craniofacial defects, all patients underwent multislice computed tomography scan (CT) according to the protocol of the CTI (Figures 2(a)–2(c)). Then, these images were sent to the CTI for subsequent fabrication of the 3D craniofacial prototype.

With the aid of the prototype, custom-made PMMA implants were fabricated preoperatively. Since none of the patients presented with functional defects of the frontal sinus, the proposed reconstructive surgeries were intended primarily to recreate the premorbid anatomical and morphological craniofacial contours.

**Figure 3:**
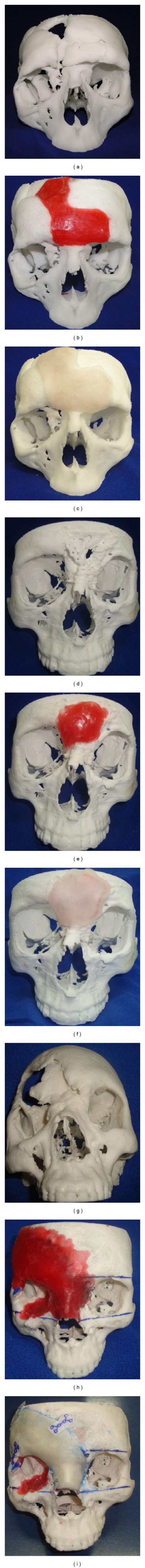
Preoperative customization of PMMA implants: initial aspect of the defect, wax covering the defects with a thickness-reduced wax, and PMMA reconstruction of the defect according to the desired anatomical contour (Case 1 ((a)–(c)), Case 2 ((d)–(f)), and Case 3 ((g)–(i))).

The prototypes were reconstructed with modeling wax at the defective regions of the skull to accurately simulate the morphology of the PMMA implants. Then, the thickness of the wax was reduced by approximately three millimeters, which allowed all involved areas to receive insulating liquid. This liquid allowed the PMMA material to be poured over these areas during its plastic phase, in accordance with the desired anatomical contour, with lateral small edge-shaped extensions, to provide support over healthy bone. After polymerization of the PMMA, the implant was polished and underwent ethylene oxide sterilization (Figures 3(a)–3(i)).

### 2.5. Surgical Technique

**Figure 4:**
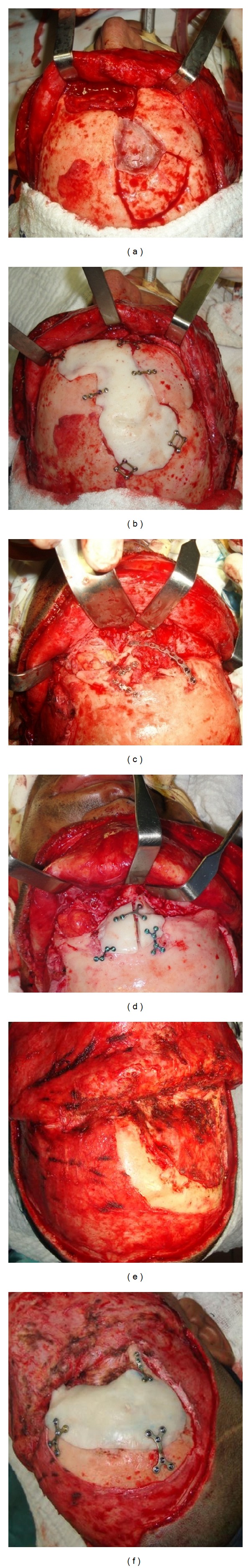
Intraoperative view of the defect and reconstruction with the PMMA implant (Case 1 ((a)-(b)), Case 2 ((c)-(d)), and Case 3 ((e)-(f))).

All surgical procedures were performed under general anesthesia, with access to the craniofacial defect(s) with a coronal approach, providing adequate exposure of the affected region(s). After careful debridement of the osseous defects, with removal of soft tissue fibrous ingrowth and small bone irregularities, the PMMA implants were adapted and fixated to the facial skeleton with titanium plates and screws. In all cases, the PMMA implants fit precisely and passively at the craniofacial defects, requiring only minor corrections to provide better adaptation (Figures 4(a)–4(f)). None of the patients had their frontal sinus approached, since no preoperative sinus complaints were reported or observed.

**Figure 5:**
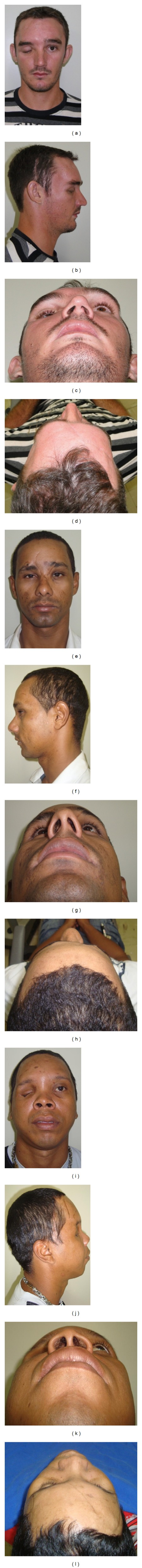
Clinical postoperative evaluation.

Postoperative objective and subjective patient evaluation demonstrated uneventful recovery and an excellent aesthetic result (Figures 5(a)–5(l)).

## 3. Discussion

Frontal bone fractures comprise about 2% to 15% of all facial fractures. The goals of craniofacial fracture treatment are isolation of the intracranial contents, treatment of any associated cerebrospinal fluid leak, and functional, as well as aesthetic, restoration of the original bone anatomy and contour [2]. Although it is not the purpose of this paper, it is important to note that upper facial reconstruction may involve management of the frontal sinus since the frontonasal duct might be impaired as a result of the craniofacial defect. Also, management of frontal sinus posterior wall fractures might indicate the need for frontal sinus obliteration or cranialization [2]. When a decompressive craniotomy is performed, the bone fragment removed from the skull may be banked and used to reconstruct the defect later. For this, the fragment might be stored in the abdominal wall and then frozen in storage [1]. However, such techniques may have the disadvantages such as resorption of the bone graft or infection [4, 7]. Patients undergoing decompressive craniotomy can develop the “syndrome of the trephined,” with symptoms such as dizziness, anxiety, irritability, and intolerance to noise or vibration. The reconstruction of cranial bone defects has proven to be effective in the treatment of this syndrome [4, 8]. This reinforces the concept that reconstructive procedures are intended for not only aesthetic restoration but also functional rehabilitation.

The use of autogenous bone remains the first choice for reconstruction of bone defects, and this may include corticocancellous bone or vascularized bone flaps that may also provide a soft tissue pedicle for reconstruction of significant soft tissue defects. However, disadvantages include the risk of bone graft resorption, insufficient availability of the required quantity of bone, or the refusal of the patient to approach another surgical site for harvest of the bone graft due to the associated morbidity. These factors would tend to favor the use of an alloplastic material [2, 3, 5–7, 9]. Advantages of alloplastic materials include ready availability, resistance to resorption, easy handling and workability during surgical procedures, and long-term stable and satisfactory cosmetic results [3, 5, 7]. The materials used most commonly are hydroxyapatite cement, hydroxyapatite block, hydroxyapatite granules, carbonated calcium phosphate bone cement, calcium phosphate paste of calcium carbonate, carbonated calcium phosphate plate, titanium mesh, high-density porous polyethylene implants, bioactive glass ceramic implants, polyetheretherketone, and acrylic materials (PMMA) [2, 3, 5].

The use of titanium mesh is limited due to its high cost and the difficulty to contour and model the material, and it is also time consuming to perform these adjustments at the time of reconstructive surgery [5, 9]. The use of demineralized bone allograft material is promising but still carries the risk of resorption, immunologic reactions, and transmission of infectious disease, thus limiting its widespread use. Although hydroxyapatite is becoming increasingly popular due to its biocompatibility and possible osteoconductive and osteoinductive properties, when compared to PMMA, the latter is less expensive, more readily accessible, and easier to handle and contour for specific craniofacial defects [9].

Currently, the alloplastic material mostly used by surgeons for these craniomaxillofacial reconstructions is PMMA, which is composed of fine particles of prepolymerized resin mixed with methyl methacrylate [1–4]. The polymerization process is initiated by the reaction between the benzoyl peroxide and N-dimethyl-p-toluidine. A radiopaque material such as barium sulfite or zirconium dioxide is also added as a component of the powder. The polymerization reaction is highly exothermic, and the PMMA material may exceed 80°C. It is currently well tolerated and supports bone formation on its surface (osteoconductive). After polymerization, typically within five minutes, the PMMA releases about 3 to 5% of monomer residues and decreases to 1.2% over time. The monomer toxicity should disappear completely within four hours [3]. No monomer fragments are released after PMMA implantation, and no PMMA toxicity occurs after 48 and 78 months following surgical reconstruction [10].

Infection, foreign body reaction, and growth restriction (in skeletally immature individuals) are a few major disadvantages of the use of alloplastic materials. The risk of infection can be reduced by adding gentamicin to the acrylic mixture, using it in a sterile environment, covering the alloplast with vascularized tissues, and maintaining systemic broad-spectrum antibiotic for a period of five days after implantation [5]. To reduce the risk of foreign body reaction, the implant must be stabilized well with immobility with the use of titanium plates and screws [9]. Growth restriction in the growing patient can be prevented by avoiding the use of PMMA placed directly over craniofacial sutures in children [5]. Of course, the initial injury and subsequent scarring may be an independent risk factor for growth restriction irrespective of the use of PMMA in the reconstruction.

PMMA can be used directly during surgery, by applying it over the bony defect, while the material is in the plastic phase of polymerization. When the implant begins to polymerize, an exothermic reaction takes place, generating heat that should be controlled by cold saline irrigation. In cases of total thickness cranial reconstruction, a titanium mesh is indicated to be placed over the PMMA implant in order to protect the implant from direct trauma [9]. After polymerization occurs, the implant is removed for trimming and then replaced and fixated. Increased operative time, the exothermic reaction, contact of PMMA resin fluid with blood, and material toxicity are all disadvantages of the use of this PMMA technique [1, 2, 4]. The allergic reaction to PMMA is rare and occurs due to a reaction against a component of the PMMA, N-dimethyl-p-toluidine, which is used as an accelerator (MMA monomer). A small amount of the population is allergic to MMA, which may manifest with symptoms of stomatitis, burning mouth syndrome, or chronic hives [3].

An alternative to intraoperative placement and polymerization is prefabrication of the PMMA implant, thereby avoiding the intraoperative disadvantages and still providing excellent aesthetic results [3, 8]. With the aid of computer-planning technology, many emerging techniques provide 3D design and construction of implants using the information provided by high-definition CT images, known as computer-aided design and computer-aided manufacturing (CAD-CAM) [1, 2, 4, 6, 7]. Undoubtedly, there are many advantages with such a technique, but it should be noted that there is a higher cost when employing additional technology in the planning process.

The rapid prototyping techniques include stereolithography, fused deposition modeling, and selective laser sintering. The prototyping technology has been widely used in the industrial area but still has been used less extensively in medical and dental healthcare, mainly due to the limited number of companies providing prototyping services, the cost of this technology, and also the lack of knowledge from health professionals regarding the application of this technology, although this is improving internationally [11]. For the construction of the prefabricated PMMA implant according to the technique presented here, a rapid prototyping model, produced by stereolithography, was used to customize a structure directly from a 3D model. Rapid prototyping has been presented as a technology that favors the direct relationship between the model and the actual anatomy. This allows for surgical simulation and planning as well as preoperative customization of implants, thus reducing surgical time and allowing better communication between the surgical team and the patient [11]. Healthcare specialty areas such as orthopedic surgery, neurosurgery, maxillofacial surgery, and implantology might benefit from this technology when planning treatment of cases of trauma, pathology, and congenital or acquired craniomaxillofacial deformities.

The technique described here combines favorable factors supported by the current literature. Besides using PMMA which is widely accepted for this reconstructive purpose, the use of a rapid prototyping technique to customize a prefabricated implant without additional financial cost to patients within the Brazilian public health system was only available through a partnership with the CTI, a governmental research unit. This might not reflect public healthcare systems in other countries, which might limit their ability to provide adequate treatment for such complex cases due to financial constraints.

In large craniofacial reconstruction procedures, PMMA implants have proven to be effective, easy to handle, and biocompatible, providing excellent aesthetic and functional results. 3D reconstruction of the defect greatly assists in the diagnosis and surgical planning, allowing surgical time to be decreased. Customized prefabricated PMMA implants manufactured over the rapid prototyping models, such as those described in this study, proved to be effective and feasible. In addition to features inherent to PMMA implants, the feasibility of the technique and the access of this technology through the public health system are unique to this study.
